# Long spin lifetime and large barrier polarisation in single electron transport through a CoFe nanoparticle

**DOI:** 10.1038/srep28296

**Published:** 2016-06-22

**Authors:** R. C. Temple, M. McLaren, R. M. D. Brydson, B. J. Hickey, C. H. Marrows

**Affiliations:** 1 School of Physics and Astronomy, University of Leeds, Leeds, LS2 9JT, UK; 2 Institute for Materials Research, School of Chemical and Process Engineering, University of Leeds, Leeds, LS2 9JT, UK

## Abstract

We have investigated single electron spin transport in individual single crystal bcc Co_30_Fe_70_ nanoparticles using scanning tunnelling microscopy with a standard tungsten tip. Particles were deposited using a gas-aggregation nanoparticle source and individually addressed as asymmetric double tunnel junctions with both a vacuum and a MgO tunnel barrier. Spectroscopy measurements on the particles show a Coulomb staircase that is correlated with the measured particle size. Field emission tunnelling effects are incorporated into standard single electron theory to model the data. This formalism allows spin-dependent parameters to be determined even though the tip is not spin-polarised. The barrier spin polarisation is very high, in excess of 84%. By variation of the resistance, several orders of magnitude of the system timescale are probed, enabling us to determine the spin relaxation time on the island. It is found to be close to 10 μs, a value much longer than previously reported.

Electrical transport studies of metallic nanoparticles are at the crossover point between three-dimensional bulk materials and the behaviour of zero-dimensional quantum dots. Relatively simple single electron charging models can be applied with very high accuracy to the resulting behaviour, enabling very precise charge^1^,^2^, current^3^ and spatial^4^ metrology.

Introducing magnetic elements, either as electrodes or the particle itself, introduces spin accumulation effects into the system. It allows spin transport to be investigated at the single electron level through comparisons with modified charging theory^5^,^6^. This combination of spin transport with single electron effects is known as nanospintronics. Previous works in the field have investigated Kondo physics^7^,^8^, cotunnelling magnetoresistance enhancement^9^,^10^, anisotropic magneto-Coulomb effects^11^, spin transfer torque^12^ and enhanced spin lifetimes^13^,^14^ amongst others^15^,^16^.

One of the main conditions that must be satisfied in order to observe single electron charging effects in nanoparticle transport experiments is that the charging energy *E*_*C*_ = *e*^2^/*C* (*C* the capacitance of the island) is much greater than the thermal energy *k*_*B*_*T*. This is a less exacting requirement than for quantum dots, and the Coulomb blockade has even been observed at room temperature^17^; as well as being a useful trait for potential technological applications this means that it is not necessary to obtain sub-Kelvin temperatures to study nanospintronics. For temperatures of order tens of K, the capacitance *C* is of the order of 1 aF and therefore the particle diameter must be less than a few nanometres. Due to this small size the major experimental challenge is to isolate a single nanoparticle for study.

Routes towards fabricating single particle contacts using electron beam lithography^13^,^18^, reactive ion etching^19^,^20^, nanoindentation^11^, and advanced ion beam lithography^21^ have had some success in this. Nevertheless, scanning tunnelling microscopy (STM) is one of the most viable techniques for studying single electron charging owing to its flexibility and sub-atomic spatial resolution. Using STM it is possible to efficiently measure many particles simply by moving the tip from one to the next, simultaneously characterising size and shape and correlating this with their electronic behaviour. STM has been highly successfully applied to non-magnetic systems^22^,^23^ but there have yet been very few studies with magnetic particles^24^,^25^.

Here we use basic non-spin polarised STM to measure Coulomb staircase behaviour in ferromagnetic nanoparticles. Using existing theoretical models we are able to characterise the various tunnel barrier parameters connecting island and electrode. Capacitances, spin polarisations and spin lifetime are all determined or bounded independently. Using this method we have been able to measure a barrier spin polarisation in excess of 84% and a spin relaxation time in the nanoparticle close to 10 μs.

Figure 1a shows the basic set-up of the double magnetic tunnel junction (DMTJ) studied in this paper, including the equivalent circuit diagram. The magnetic nanoparticle is separated from a bottom magnetic Co_40_Fe_40_B_20_ (CFB) electrode by an MgO tunnel barrier, forming a nanoscale magnetic tunnel junction that is the same size as the nanoparticle. The STM tip plays an integral part in the structure, acting as a second (non-magnetic) electrode. Electrical transport characterisation is done by scanning tunnelling spectroscopy (STS). For STS the tip is held static with the feedback loop switched off, the tip bias is varied and the current through to the bottom electrode recorded. The tip/particle separation can be adjusted by manipulating the scanning feedback settings.

## Results

**Figure 1:**
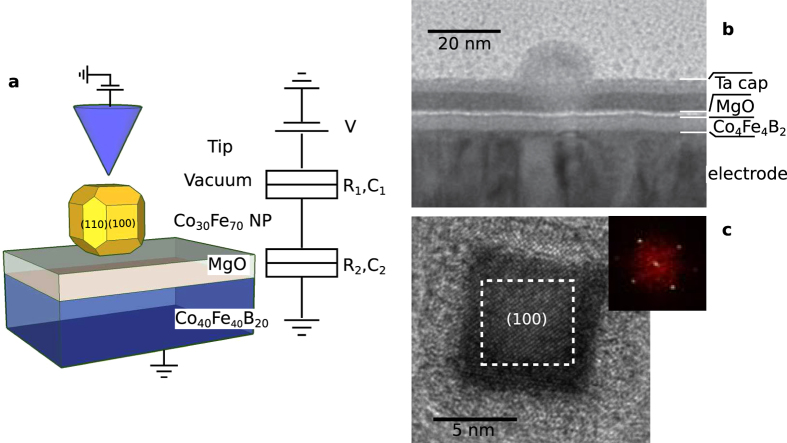
TEM planar and cross-section images of nanoparticle and half tunnel junction stack. (**a**) Diagram representing the scanning tunnelling spectroscopy experiment with equivalent circuit diagram. (**b**) Cross-section TEM image of the nanoparticle/electrode structure, a tantalum cap was used to protect this sample from oxidation during atmospheric transfer to the TEM, this cap is not included in the STM experiments. (**c**) Planar TEM of a nanoparticle with the Fourier transform of the indicated region shown right. The particle grows bcc crystalline with a 〈100〉 face perpendicular to the substrate.

Initial characterisation of the half tunnel barrier stack and nanoparticles was carried out by transmission electron microscopy (TEM). A cross-section of the growth stack is imaged in Fig. 1b. The cross-section includes a final Ta capping layer for protection during atmospheric transfer to the TEM instrument, this was not included in the samples used for the STM experiment. The MgO is seen to provide a continuous barrier layer, undeformed by deposition of the particle, this was the case in the majority of particles imaged. Planar TEM images, Fig. 1c, show that the particles have undergone Wulff reconstruction during formation, leaving twelve hexagonal 〈110〉 and four square 〈100〉 faces. The relative size of the two faces varied somewhat for different size particles. Greater than ninety percent of the particles rest with a 〈100〉 face on the tunnel barrier. This orientation is one of the conditions required for spin filtering through an MgO barrier^26^,^27^ leading to large tunnelling spin polarisation^28^,^29^.

**Figure 2:**
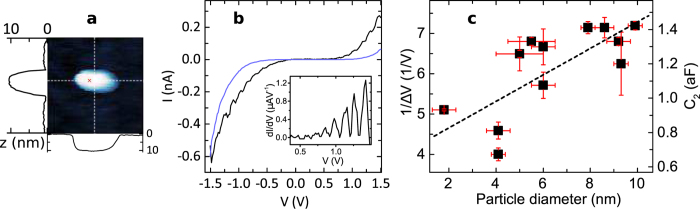
Scanning tunnelling microscopy (STM) and spectroscopy (STS) on and off nanoparticles. (**a**) A 50 × 50 nm STM topography scan of isolated 10.2 nm diameter nanoparticle. The indicated cross-section is used to determine the particle size rather than the particle area. The red × marks the point for the spectroscopy scan shown in black in (**b**), the differential of which is shown in the inset. The blue line in (**b**) is an average of several STS scans outside the particle over the MgO barrier. (**c**) Comparison of the step width Δ*V* to the particle diameter for several particles. The calculated capacitance values are shown on the right. A dashed line of best fit to the data is shown.

For the STM experiment uncapped samples were transferred in vacuum to a variable temperature STM system and cooled to approximately 22 K. Part of the advantage of the STM over point contact methods is the ability to map the topography of a particle before electrical characterisation. For each spectroscopy measurement, a topography scan was taken first. STM over an isolated particle is shown in Fig. 2a. As is typical for imaging insulators such as MgO, scanning feedback parameters require a high voltage to obtain sufficient current^30^. The image shown has feedback settings of 1.5 V and 0.4 nA. The measured particle area is large compared to the expected particle dimensions due to convolution with the tip shape, however the particle height gives a reliable particle size as shown in the profile plot.

Figure 2b displays an STS scan taken over the nanoparticle imaged in 2a at the marked point. The I-V is clearly non-linear, this is due to field emission effects as we shall discuss later. A periodically stepped gradient is convoluted with the field emission shape, this is seen more clearly in the differential data shown in the insert. In the most basic form, charging effects due to the nanoparticle reveal themselves as a Coulomb staircase in such an I-V; with each step corresponding to an integral increase in the charge state of the island. The step width is given by Δ*V* = *e*/*C*_2_ where *C* = *C*_1_ + *C*_2_ and *C*_1_ and *C*_2_ are the electrode-island and tip-island capacitances respectively^31^. The steps seen in this data are regular and indicate a capacitance *C*_2_ = 1.2 aF (the reason for taking *C*_2_ in preference to *C*_1_ here will be given later).

Imaging on an oxide barrier with large nanoparticles present was challenging. Due to the exponential sensitivity of the tip height on the current, the STS scans were somewhat inconsistent in nominally the same location. If the feedback settings were not correct it was also possible for a particle to move or simply disappear on repeated scans, likely due to tip particle interaction. To confirm that single-electron physics is indeed taking place, it was first confirmed that the periodic staircase was only seen when performing STS scans over a particle. In both measurements over particles and control experiments where spectra were taken at a position away from any particle, the tip height was set by the same bias and current feedback parameters so that the vacuum barrier was a similar width in either case.

Second we were able to check the size dependence of the step width versus the diameter of the particles. Several STS scans over particles of a range of sizes reveal an inverse correlation between Δ*V* and particle diameter shown in Fig. 2c. Approximating the electrode-island system as a parallel plate capacitor with area 36 nm^2^, dielectric thickness 1.4 nm (dimensions taken from the TEM image), and a relative dielectric constant of ~10 for MgO^32^, yields an expected *C*_2_ ≈ 2.2 aF. This is in fair agreement with the measured capacitance values. The total change in capacitance with respect to the diameter in the range shown is not as high as would be expected however, this could be due to a combination of the simplicity of the model and inaccuracies in measuring the height of the smallest particles relative to the STM noise base.

In order to characterise the junction beyond the capacitance *C*_2_, a full fit to the orthodox single electron theory is required. The current in a basic double tunnel junction with no spin accumulation, under the assumption of asymmetric junction resistances 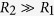 has a basic staircase form that can be written analytically as^31^,^33^:


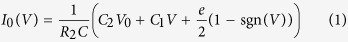


with *Ne* the integral charge state of the island, sgn a function that returns −1 (+1) for a negative (positive) value input, 
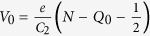
 the bias at the start of each step, and *Q*_0_*e* the excess static charge of the island due to stray environmental charge (charged defects in the oxide barrier, for example). This staircase function tends towards a linear relationship for the case of *C*_1_ > *C*_2_. We will see later that the ratio of the resistances of the two barriers is very large (greater than 10^3^). There are two possibilities therefore: the resistance of the vacuum barrier is very large and the capacitance is small or the resistance is much smaller than the MgO barrier and the capacitance is large. The MgO dielectric means that in our system *C*_2_ is greater than *C*_1_. We can therefore infer that MgO barrier resistance *R*_2_ is much greater than the vacuum resistance *R*_1_. The MgO barrier is therefore the rate controller in this set-up, and the step width is given by *e*/*C*_2_. The model is not sensitive to *R*_1_ in this case so that exact knowledge of the tip height is not required.

**Figure 3:**
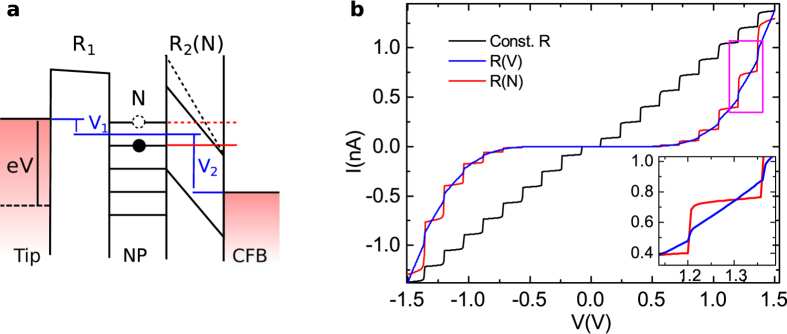
Field emission modified Coulomb staircase model and simulation. (**a**) Diagrammatic representation of the model. *V* increases smoothly while *V*_2_ shows discrete jumps as new energy levels become available. (**b**) The various models plotted for a typical parameter set, see text for full description.

The model above describes a simple linear staircase plotted in black in Fig. 3b. This is not sufficient to fit to the non-linear staircase observed in Fig. 2b. This is due to an assumption that the effective tunnelling resistance *R*_2_ is constant with applied bias. In fact, due to the field emission effect at these relatively high voltages, the resistance is changing exponentially with *V*.

This is a common issue in fitting such STM data and various corrections have been applied in the past in order to reconcile the model to the data^22^,^34^. A naive route is to simply replace


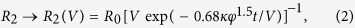


according to the 1D tunnelling Simmons model^35^. Here *κ* is the evanescent wavevector, *φ* the barrier height, *t* the barrier thickness and *R*_0_ a constant. This model is shown in blue in Fig. 3b and gives an exponential current background. In this case, however, the staircase is washed out nearly completely at large applied bias. This is contrary to what we see in the data where the steps are still prominent at large applied bias.

To solve this problem we consider the energy diagram shown in Fig. 3a. Due to the bottleneck of resistance at the MgO barrier (*R*_2_) the majority of the applied bias is dropped across the MgO barrier. This is contrary to the double barrier situation present when no particle is scanned, in this case the dielectric forces a large electric field to be present in the vacuum barrier. We see further that the electric field will not be continuous with increasing *V* but in fact increase stepwise according to the state of the island *N*. We therefore replace *R*_2_ → *R*_2_(*N*) = *R*_2_(*V*_2_) in our model equations, where now *V*_2_ is the bias drop across the MgO barrier. The simulated curve under these approximations is plotted in red in Fig. 3b. The step tops are now flattened, since while the island is in state *N* the electric field across the barrier does not change as a function of *V*. This approach broadly follows the theory of Raichev^36^. The theory has been experimentally confirmed by a few groups^37^,^38^,^39^, however the data presented here are probably the clearest example of this type of tunnelling in a single metallic particle to date. More complicated effects predicted due to electric charge distribution in the nanoparticle are not observed here^36^.

While the *R*(*N*) field emission model was able to fit spectra with at most a flat step top, it was observed that many of the spectra recorded on the full DMTJ structure showed further reduced or negative gradient within the steps. This negative gradient and peak step structures can only be modelled through spin accumulation effects.

The approximate form of *I* for the first part of each step under spin accumulation is given by^33^:


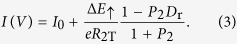


The spin accumulation on the particle modifies the spin dependent chemical potential Δ*E*_*σ*_ (*σ* ∈ ↓, ↑) on the island. The accumulation rate is governed by the spin resistance ratio on either side of the barrier *P*_*i*_ = *R*_*i*↑_/*R*_*i*↓_ (*i* ∈ 1,2), and the ratio of the spin dependent density of states at the Fermi energy 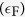 on the particle 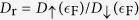. These split energy levels lead to overall changes in the conductance which manifest as peaks or troughs in the staircase.

To generate independent fits of the various barrier parameters, the structure of the data is broken down into several elements dependent on just one or two parameters. Each parameter was calculated or fitted individually using eq. (3) before moving onto subsequent features. The analytical form (3) was used as a guide to the starting parameter set for the full numerical solution (see methods section for details). This modular approach was found to be necessary due to the computational resources required to generate a simulated spectrum for each parameter set and the complexity of the data.

**Figure 4:**
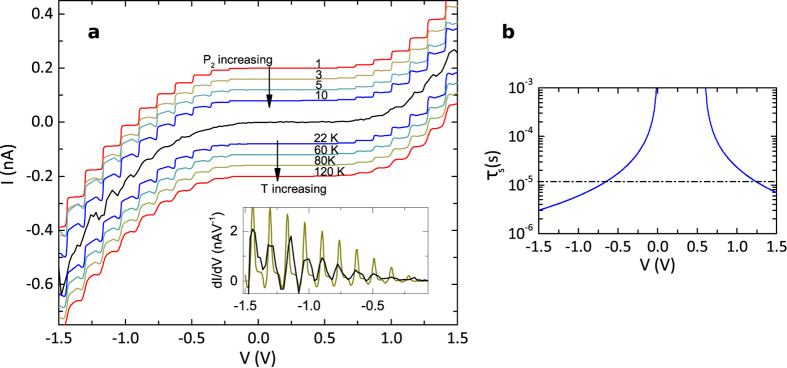
Spin accumulation fit to STS data and spin lifetime analysis. (**a**) Experimental STS data shown in black and compared to curves calculated from spin accumulation charging theory with various fit parameters. The top set of calculated curves, from red to blue, map the increasing spin polarisation of the tunnel barrier, the bottom set of calculated curves vary the temperature as listed while maintaining *P*_2_ = 10. The calculated curves are offset so that their details may be discerned. The inset shows the differential plots of the experimental data and the best fit data at 80 K. The full parameter set is described in the text. (**b**) The approximate probed system timescale as a function of applied bias. The dashed line indicates the implied spin relaxation time on the island.

Figure 4a shows a Coulomb staircase I-V spectrum for a particle of ~10 nm diameter, along with a selection of calculated curves in which the system parameters are adjusted to find the closest resemblance to the measurement. The simplest feature to fit first is the period and offset of the steps, these are governed by *C*_2_ and *Q*_0_. These parameters are determined the most accurately and in this case take the values *C*_2_ = (1.09 ± 0.01) aF and *Q*_0_ = (0.1 ± 0.05)*e*.

Next the overall exponential profile of the curve is fitted to eq. (2) to find *R*_2*T*_(*V*_2_). Here *V*_2_ depends on *N* as discussed above. Due to the asymmetric barrier, the fitting parameters found were different for positive and negative bias. For the negative (positive) side *dφ*^1.5^ was found to be 0.76 ± 0.01 Å eV^1.5^ (4.30 ± 0.01 Å eV^1.5^).

The remaining spin-independent parameters, *R*_1_ and *C*_1_, govern the internal shape of the step. In this case, after removal of the spin component the step top is found to be flat. This indicates a highly asymmetric capacitance ratio of *C*_2_/*C*_1_ > 10. This is to be expected, since the asymmetry is due to the relative dielectric constants of the MgO and vacuum barriers. Similarly, a flat step indicates a large asymmetric resistance ratio of *R*_2*T*_/*R*_1*T*_ > 10^3^. These are the ideal conditions for observing a Coulomb staircase, conveniently realised naturally in the asymmetric set-up of the STM double tunnel junction. A calculated curve using the spin independent parameters generated so far is shown as the top red line of Fig. 4, in which the steps are flat.

The STM tip material is a non-magnetic material, tungsten, so that the spin resistance ratio for the vacuum barrier should purely depend on the island density of states ratio *D*_r_. We therefore limit solutions to *P*_1_ = 1/*D*_r_, leaving two independent spin parameters *P*_1_ and *P*_2_. The top series of calculated curves in Fig. 4 show increasing *P*_2_ from red to blue as the spin dependent tunnelling is gradually turned on. The crucial point is that the peak feature emerges, so that the step top is no longer flat and there is a region of negative differential conductance in each step of the staircase, which is the signature of spin dependent tunnelling. The blue line (*P*_2_ = 10) shows the closest resemblance to the relevant features of the measured STS spectrum. In fact the gradient decrease saturates for *P*_2_ > 15 so that it is not possible to put an upper bound on the value. The spin polarisation of the current through the bottom barrier oscillates with applied bias but peaks according this fit at 84–100%. The fit is relatively insensitive to *P*_1_ at these large values of *P*_2_. The model is satisfied for all values of 0 < *P*_1_ < 0.6, or equivalently *D*_↑_/*D*_↓_ > 1.7.

In these samples the CFB/MgO spin filter layers have not been heated above room temperature. Annealing encourages the (100) crystalline texture which is required for the highest TMR and spin polarisation ratios^26^; nevertheless we have measured a relatively high spin polarisation. Previous measurements on unnannealed patterned MTJ junctions have found a lower spin polarisation of 20%^40^. The CFB layer is inevitably somewhat granular when grown by sputter deposition, measurements on patterned junctions are an average over many grains. Here the nanoparticle is interacting with just a few grains, so we may expect a higher spin polarisation within the grain area. Earlier work studying CFB/MgO layers using STM found that the necessary crystalline texture exists within the grains without annealing^30^. Annealing in a field is also required for out-of-plane anisotropy, we therefore have an in-plane anisotropy of the CFB layer. The anisotropy of the nanoparticle does not have the same shape-anisotropy limitation and its magnetisation direction is unknown. To create such a high spin filter however it is likely that some component of the particle magnetisation is anti-parallel to the film magnetisation.

It is perhaps surprising that *R*_1↓_ > *R*_1↑_, whereas *R*_2↓_ < *R*_2↑_, given the common magnetic state of the island that the barriers share. The vacuum barrier is the simpler of the two junctions, joining a tungsten tip to the nanoparticle, and probably follows a Jullière-type tunnelling model most closely. The MgO junction is more complicated due to the materials present, joining a crystalline Co_30_ Fe_70_ nanoparticle to an amorphous CFB underlayer through a polycrystalline barrier. The junction polarisation is sensitively dependent on the barrier details and interface electronic states and in this case evidently has a negative value^41^. A more accurate study of these surface states would allow more detailed theoretical calculations on this particular barrier arrangement.

Throughout the modelling the temperature *T* has been held at the experimentally measured value of 22 K. In this experiment however, only the sample temperature was controlled via a cold finger insert. The temperature of the tip was not regulated and is likely to be much warmer. The tunnelling rate is far more sensitive to the Fermi distributions at the least resistive tunnel barrier, in this case the tip-island gap, and so we may expect to need to make some correction for this. The calculated I-V spectra shown in the bottom part of Fig. 4 are for increasing temperature, from blue (22 K) to red (120 K), these calculated spectra retain all other fit parameters from the *P*_2_ = 10 fit shown above in blue above the data. The best fit to the data is obtained for a temperature of 80 K, indicating a tip electron temperature close to this value. In this case the step riser is broadened somewhat. The corresponding fit to the measured differential conductance is shown in the inset. The height and width of the differential peaks matches the data far better at this temperature. The presence of negative differential conductance on each step, due to spin-dependent tunnelling, is very clearly seen when the data are presented this way.

Finally we come to the spin relaxation time on the island. Since significant spin accumulation effects have been observed, the spin relaxation time on the island *τ*_r_ must far exceed the characteristic tunnelling rate *τ*_s_ = *e*^2^*R*/*δ*, where *δ* is the energy spacing of the island states at the Fermi energy^33^,^42^. This can be intuitively understood as being roughly the average time an electron spends on the island as it tunnels through, the spin coherence time must exceed this in order for effects to be observed. Using the particle diameter of 10 nm, a density of states of *D*_↑_ ≈ 0.25 eV^−1^atom^−1^^43^ and the resistance at the larger applied biases *R* = 2.5 GΩ, we find *τ*_s_ ≈ 2 μs.

While the above analysis gives a lower limit on the spin relaxation time of the island *τ*_r_, the only way to quantify *τ*_r_ by this method is to have a characteristic system timescale *τ*_s_ that is similar to the relaxation timescale. Previous studies have been unable to pin down the spin lifetime accurately due to the difficulty of controlling *τ*_s_ experimentally. In the present study however, due to the field emission, the effective tunnel barrier resistance is varying exponentially with applied bias. Figure 4b shows the calculated *τ*_s_, which is linearly dependent on the barrier resistance, as a function of applied bias. *τ*_s_ is conveniently varied over several orders of magnitude. Looking at the spectroscopy data in 4**a** we see the spin accumulation effects on the Coulomb staircase are reduced between −0.8 V and 1.2 V, these intersections are indicated as a dashed line on Fig. 4b. The spin relaxation time on the island *τ*_r_ is extracted as approximately 10 μs.

## Discussion

This relaxation time compares with a bulk material *τ*_r_ ≈ 30 fs^44^ and previous studies on metallic nanoparticles of 150 ns in Co particles^13^ and 1 μs at low voltages in Al particles^45^. While the spin lifetime found in this paper is somewhat approximate due to the relatively small signal observed, the order of magnitude is significant. The massively extended spin lifetime in nanoparticles is due to reduced spin-flip scattering. Mechanisms suggested previously for this have included quantised energy levels suppressing spin-orbit induced spin-flip scattering rates, and reduced magnon scattering due to quantised magnon energy^13^,^14^,^19^. Both of these effects have comparable energy scales in these particles^19^ and are likely to contribute to the lifetime enhancement. Future detailed studies based on the technique described here could yield significant insight into the exact nature of this phenomenon; in particular the temperature dependence would be of interest.

In conclusion we have investigated spin dependent transport through an individual Co_30_Fe_70_ nanoparticle using scanning tunnelling spectroscopy. The I-V spectra were seen to exhibit Coulomb staircase features, the voltage spacing of which were correlated to the size of the island. It was shown for the first time in this system that the data could be modelled using orthodox Coulomb blockade theory modified to allow for discrete bias dependent resistance, a common requirement for STM experiments of this type. Simulation of all the salient features of the Coulomb staircase data using the full model including spin accumulation effects was demonstrated. The spin ratio *P*_2_ of the MgO tunnel barrier was found to be greater than 15, indicating a peak spin current polarisation in excess of 84%. It was seen to oppose the spin density of states ratio of the bulk nanoparticle and this was attributed to the strong dependence of the tunnelling on the barrier interface states. Finally the spin lifetime on the island was discussed. Unusually due to the field emission effects it was possible to vary the characteristic tunnelling rate over several orders of magnitude, the spin relaxation rate was found to be close to 10 μs. This demonstrates the predicted potential for external field-free tunnel barrier characterisation using the detailed features of the spin dependent Coulomb staircase. The large tunnelling spin polarisation and very long spin relaxation time makes such systems appealing for nanospintronic applications.

## Methods

### Growth and characterisation

The bottom magnetic electrode, tunnel barrier and nanoparticles were first deposited onto thermally oxidised single-polished Si wafer. The electrode and barrier were grown by DC and RF sputtering respectively, the growth details are given elsewhere^46^ but the final termination layers were Co_40_Fe_40_B_20_ (4 nm)/MgO (1 nm). An *in-situ* Oxford Applied Research gas aggregation nanoparticle source was used to deposit preformed bcc Co_30_Fe_70_ nanoparticles onto the surface in the 2–12 nm diameter regime, for further details of this technique see refs 47,48. A nominal particle density of ~100 μm^2^ was grown, allowing for individual particle imaging. The base pressure of the sputter chamber was 8 × 10^−6^ Pa. The particles were deposited at less than 0.1 eV/atom and are not deformed on impact with the barrier.

Subsequent to sputter deposition the specimen was transferred via portable vacuum chamber at 10^−7^ Pa to an Omicron variable temperature STM held at pressure of 10^−8^ Pa. X-ray absorption spectroscopy studies of equivalent capped particles grown under the same conditions showed no trace of an oxide shell formed on the particles^48^. Scanning tunnelling microscopy (STM) and spectroscopy (STS) were carried out at the base temperature of the instrument of approximately 22 K. The cooling was enacted through a cold finger insert clamped to the sample plate via a brass block. The tip used was chemically etched tungsten, annealed *in-situ* following a standard process^49^.

TEM measurements were undertaken on a nominally equivalent sample which included a final Ta capping layer for protection during atmospheric transfer to the TEM instrument. Planar and cross-sectional sample sections were imaged at 200 kV using a Technai TF20 TEM/STEM. The cross section of the sample was generated using a cut and dimple grind process on a “sandwich” specimen in a slotted rod, followed by ion polishing using a Gatan precision ion polishing system (PIPS) model 691.

### Orthodox single electron charging theory accounting for spin accumulation

To model the data we follow the standard notation and orthodox theory of single electron physics^5^,^6^,^33^. To summarise, the current is given by an incoherent sum of tunnelling rates over Fock space states with the quantum number *N* being the number of excess electrons on the island.


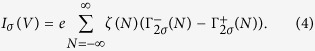


The probability *ζ*(*N*) of the island to be in state *N* is governed by a master equation dependent on the tunnelling rates onto (+) and off (−) either side of the particle. The tunnelling rates 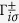 (*i* is 1 or 2 for the tip-island and electrode-island barriers respectively) are given by


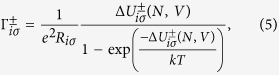


where the effective tunnelling resistance *R* crucially depends on the spin state *σ* (↑ or ↓) of the electron that is tunnelling. Δ*U* is the energy change due to the tunnelling event and has the form


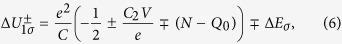


and similarly for Δ*U*_2_. Finally, the chemical potential energy change Δ*E*_*σ*_ on the island due to spin accumulation is governed by spin conservation:


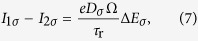


where Ω is the particle volume, *D*_*σ*_ is the island density of states at the Fermi energy for electrons of spin *σ*, and 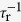 is the spin relaxation rate on the island. Charge conservation relates the down spin accumulation as *D*_↑_Δ*E*_↑_ = −*D*_↓_Δ*E*_↓_.

### Data Availability

Data associated with this work are available from the Research Data Leeds repository under a CC-BY license at http://doi.org/10.5518/82.

## Additional Information

**How to cite this article**: Temple, R. C. *et al*. Long spin lifetime and large barrier polarisation in single electron transport through a CoFe nanoparticle. *Sci. Rep.* **6**, 28296; doi: 10.1038/srep28296 (2016).
